# Plasma Soluble (Pro)renin Receptor Reflects Renal Damage

**DOI:** 10.1371/journal.pone.0156165

**Published:** 2016-05-26

**Authors:** Naro Ohashi, Shinsuke Isobe, Sayaka Ishigaki, Takahisa Suzuki, Takamasa Iwakura, Masafumi Ono, Tomoyuki Fujikura, Takayuki Tsuji, Atsushi Otsuka, Yasuo Ishii, Hiroshi Furuse, Akihiko Kato, Seiichiro Ozono, Hideo Yasuda

**Affiliations:** 1 Internal Medicine 1, Hamamatsu University School of Medicine, 1-20-1 Handayama, Higashi-ku, Hamamatsu 431–3192, Japan; 2 Urology, Hamamatsu University School of Medicine, 1-20-1 Handayama, Higashi-ku, Hamamatsu 431–3192, Japan; 3 Blood Purification Unit, Hamamatsu University School of Medicine, 1-20-1 Handayama, Higashi-ku, Hamamatsu 431–3192, Japan; University Medical Center Utrecht, NETHERLANDS

## Abstract

**Background:**

(Pro)renin receptor [(P)RR], a specific receptor for renin and prorenin, was identified as a member of the renin-angiotensin system (RAS). (P)RR is cleaved by furin, and soluble (P)RR [s(P)RR] is secreted into the extracellular space. Previous reports have indicated that plasma s(P)RR levels show a significant positive relationship with urinary protein levels, which represent renal damage. However, it is not fully known whether plasma s(P)RR reflects renal damage.

**Methods:**

We recruited 25 patients who were admitted to our hospital to undergo heminephrectomy. Plasma s(P)RR levels were examined from blood samples drawn before nephrectomy. The extent of renal damage was evaluated by the levels of tubulointerstitial fibrosis. Immunohistochemical analysis of intrarenal (P)RR and cell surface markers (cluster of differentiation [CD]3, CD19, and CD68) was performed on samples taken from the removed kidney. Moreover, double staining of (P)RR and cell surface markers was also performed.

**Results:**

There were significant positive relationships between plasma s(P)RR and tubulointerstitial fibrosis in all the patients and those not receiving RAS blocker therapy. Significant positive relationships were found between plasma s(P)RR levels and the extent of tubulointerstitial fibrosis after adjustment for age, sex, body weight, blood pressure, and plasma angiotensin II, in all the patients and those not receiving RAS blockers. Moreover, (P)RR expression was elevated in infiltrated mononuclear cells but not connecting tubules or collecting ducts and vessels. Infiltrated cells positive for (P)RR consisted of CD3 and CD68 but not CD19.

**Conclusions:**

These data suggest that plasma s(P)RR levels may reflect (P)RR expression levels in infiltrated mononuclear cells, which can be a surrogate marker of renal damage.

## Introduction

(Pro)renin receptor [(P)RR] is a specific receptor for renin and prorenin and has been identified as a component of the renin-angiotensin system (RAS) by Nguyen et al. in 2002 [1]. (P)RR is a 350-amino acid protein with a single transmembrane domain and is widely expressed in various tissues, including the kidney, heart, liver, placenta, and pancreas. (P)RR is especially expressed in various regions of the kidney, including glomeruli (mesangial cells and podocytes), proximal and distal tubules, collecting ducts, and vessels [1–3].

When prorenin binds to (P)RR, multifunctions are fulfilled. First, prorenin undergoes a conformational change, without proteolytic cleavage, to develop renin activity. This process plays a role in tissue RAS regulation. Second, (P)RR activation triggers the mitogen-activated protein kinases and extracellular signal-regulated kinase 1/2 phosphorylation, which in turn upregulates the expression of profibrotic genes such as transforming growth factor-β 1, plasminogen activator inhibitor type 1, collagens, and fibronectin. In addition, (P)RR functions as an accessory protein of vacuolar proton adenosine triphosphatase (V-ATPase) and is involved in the control of intracellular and extracellular pH. Finally, (P)RR is associated with Wnt-β-catenin signaling pathways, which are essential for adult and embryonic stem cell biology, embryonic development, and diseases such as cancer [4, 5].

Cleavage of PRR by furin yields soluble (P)RR [s(P)RR]. Thereafter, s(P)RR is secreted into the extracellular space and is ultimately found in blood and urine [3, 6]. Because s(P)RR also binds to prorenin and mediates the activation of prorenin, it is possible that s(P)RR also has physiological functions [3].

The importance of s(P)RR has been indicated in clinical studies. Watanabe et al. demonstrated that high circulating levels of s(P)RR during early pregnancy predict a subsequent elevation in blood pressure (BP), and high concentrations at delivery were associated with preeclampsia [7]. Moreover, in patients with chronic kidney disease (CKD), Hamada et al. recruited 374 patients whose mean serum creatinine (sCr) levels were 1.9 ± 1.6 mg/dL. In their study, they demonstrated that s(P)RR levels were positively associated with levels of urinary protein/urinary Cr. In addition, they indicated that the baseline levels of s(P)RR were positively associated with the annual change of sCr, and that those were negatively associated with the annual change of estimated glomerular filtration rate (eGFR) [8]. However, there was no direct evidence as to whether plasma s(P)RR levels were associated with renal damage. Therefore, this study was performed to clarify the relationships among plasma s(P)RR and renal damage using kidney samples that were obtained by nephrectomy.

## Materials and Methods

### Recruitment of patients

This study was approved by the ethics committee of Hamamatsu University School of Medicine (No. 25–92) and was conducted in accordance with the guidelines of the Declaration of Helsinki. All the patients provided written informed consent. We recruited 25 patients (age range, 20–80 years) who were admitted to our hospital to undergo heminephrectomy between September 2013 and July 2015, regardless of the range of renal function and antihypertensive medication. The causes of nephrectomy were the following: renal cell carcinoma, 22; ureteral carcinoma, 2; and kidney metastasis, 1.

### Study protocols

In patients on hemodialysis (HD), vital signs, such as height, dry weight, systolic and diastolic BPs, and heart rate, were measured on the day before surgery. The HD session was performed after the measurements. Blood samples were drawn just before the HD session. Both patients on peritoneal dialysis and not on dialysis also underwent the aforementioned procedures preoperatively. The procedures were performed 0.88 ± 0.99 days before nephrectomy.

The patients were asked to rest in the supine position for at least 15 min before blood sample collection at 9 am. Thereafter, blood samples were centrifuged at 3000 rpm for 10 min at 4°C and stored at −80°C until further assay as per a previous report [9]. Kidney samples were collected immediately after nephrectomy and prepared as described in the subsequent sections.

### Measurement of renal function and plasma renin activity (PRA), plasma angiotensin II (AngII) and plasma s(P)RR

The sCr concentrations were measured in the clinical laboratory of the study hospital.

The eGFR was calculated using the Japanese eGFR equation [10]. PRA and plasma AngII levels were determined by radioimmunoassay (RIA) (SRL, Tokyo, Japan) and plasma s(P)RR level were measured by enzyme-linked immunosorbent assay (ELISA) according to the manufacture’s instruction (IBL, Takasaki, Japan).

### Evaluation of tubulointerstitial lesions

A small part of the resected kidney was fixed in formalin and embedded in paraffin.

Tissue sections (3 μm) were stained with Masson’s trichrome for histopathological evaluation of tubulointerstitial lesions. Because the extent of tubulointerstitial fibrosis reflects the severity of renal damage [11], the percentages of tubulointerstitial fibrosis were evaluated in microscopic fields observed at ×100 magnification. Ten microscopic fields were evaluated for each patient using a point-counting method, and mean values were calculated. All quantitative analyses were performed in a blind manner to avoid bias.

### Immunohistochemical analysis of intrarenal (P)RR

To evaluate the intrarenal (P)RR expression regions and levels, immunostaining for (P)RR in kidney sections was performed using the EnVision^TM^ + Dual Link System-HRP (Dakocytomation, Glostrup, Denmark), as previously described [11–14]. The primary antibody was rabbit anti-(P)RR antibody, which detected within residues 300 to the C-terminus (Abcam, Tokyo, Japan). Sections incubated without the primary antibody were used as controls. To examine the expression levels of (P)RR, 10 small vessels and collecting ducts or connecting tubular cells and infiltrated cells in 10 microscopic fields at ×100 magnification were evaluated for representative samples according to the stained levels for (P)RR: unstained, 0; weak, 1; moderate, 2; and severe, 3, and mean values were calculated.

### Immunohistochemical analysis of infiltrated cells using cell surface markers in serial sections

Infiltrated cells were stained by using cell surface markers to determine what kinds of cells were infiltrated. CD3-, CD19-, or CD68-positive cells indicate T cell, B cell, and monocyte/macrophage lines, respectively. The primary antibody for CD3 was mouse monoclonal from Novocastra/Leica Biosystems, Heiderberg, Germany, and those for CD19 and CD68 were mouse monoclonal from Dakocytomation. The patient with eGFR of 27 mL/min/1.73m^2^ was selected for immunohistochemical analyses because remarkable infiltrated cells were present in the tissue section.

### Double staining by immunofluorescence

Double staining of paraffin sections was performed using anti-(P)RR antibody with either anti-CD3, anti-CD19, or anti-CD68 antibody. The sections were incubated with Alexa Fluor 546- and 488-labeled secondary antibodies (Molecular Probes, Eugene, OR, USA) for the anti-(P)RR and cell surface marker antibodies, respectively. The patient whose eGFR was 27 mL/min/1.73m^2^ was selected for the immunofluorescence analyses. Localization of (P)RR and cell surface markers was investigated with an immunofluorescence microscope (BX50, Olympus, Tokyo, Japan).

### Statistical analysis

Results were expressed as the mean ± standard deviation. Dry weight was used as body weight for analyses in patients on HD. All parameters, except for PRA, showed a normal distribution. On the other hand, because PRA did not show a normal distribution, logarithmic transformation was applied. The correlations between plasma s(P)RR and other parameters were evaluated by using Pearson’s product-moment correlation coefficient. Multiple linear regression analyses for plasma s(P)RR levels were adjusted for age, sex, body weight, systolic BP, plasma AngII, and the extent of interstitial fibrosis. Age, sex, and body weight were selected as variables because these parameters are common in performing multiple linear regression analyses. In addition, systolic BP and plasma AngII, component of circulating RAS, were also adjusted as they are associated with renal damage. We considered p < 0.05 statistically significant. Statistical analyses were performed using Statistical Package for the Social Sciences (SPSS) software version 20 (SPSS Inc., Chicago, Illinois, USA).

## Results

### Characteristics of all the patients

**Fig 1:**
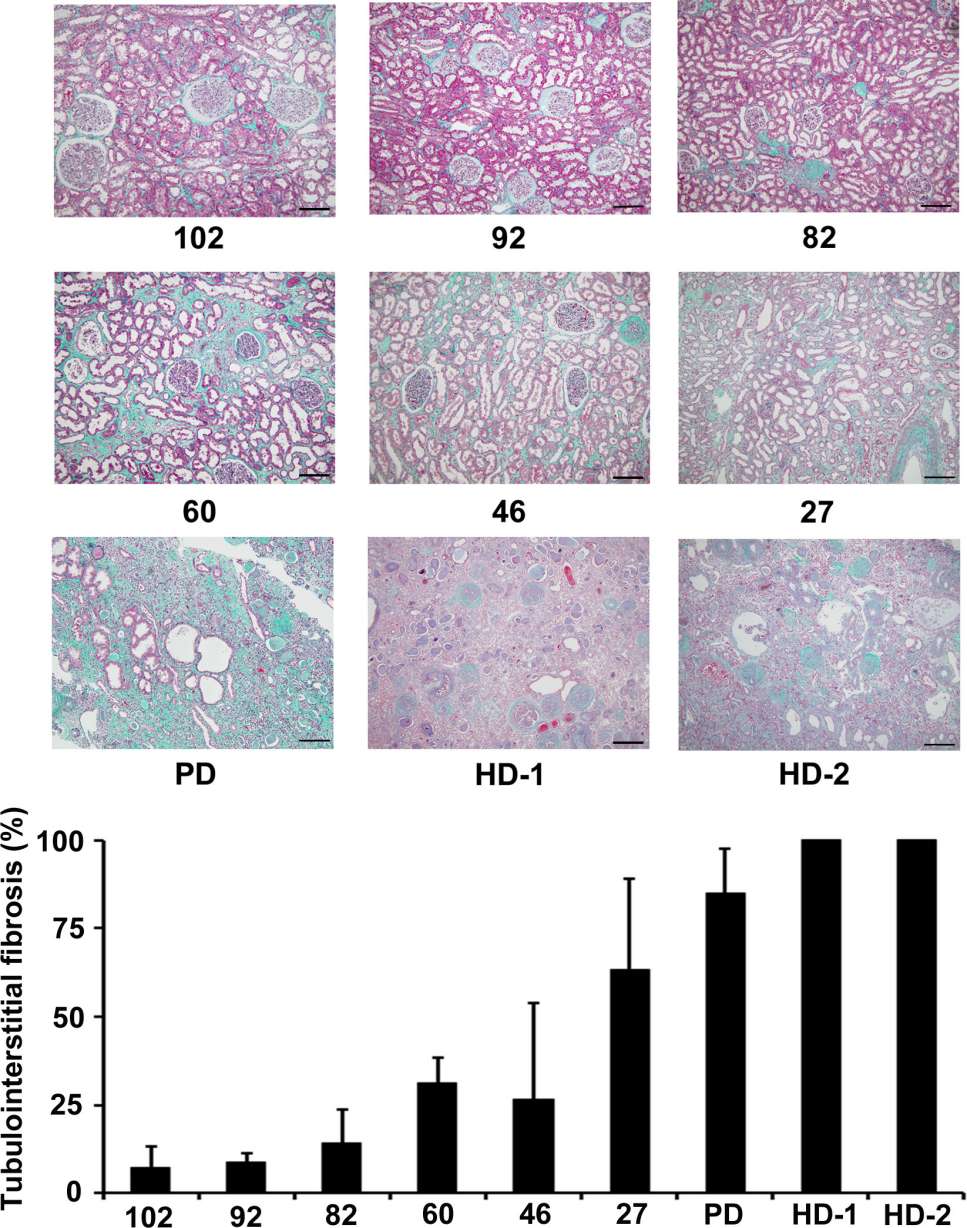
Tubulointerstitial damage of the patients with all the range of renal function. Masson’s trichrome staining was performed for histopathological evaluation of tubulointerstitial damage. Numbers below each figure mean estimated glomerular filtration rate of each patient. Original magnification ×100. The scale bar in each figure represents 100 μm. Patients who were representative for the stages of chronic kidney disease were selected at random. The graph indicates the percentages of tubulointerstitial fibrosis that were evaluated in microscopic fields observed at ×100 magnification. Ten microscopic fields were evaluated for each patient using a point-counting method, and mean values were calculated. Abbreviations: PD, peritoneal dialysis; HD, hemodialysis.

The patient characteristics are listed in Table 1. Dialysis was performed for all the patients with an eGFR < 15 mL/min/1.73m^2^; 8 patients required HD and 1 required PD. The extent of tubulointerstitial damage was proportional to the extent of renal dysfunction (Fig 1).

**Table 1:** Characteristics of all the patients.

Age, year	62.9 ± 15.0
Sex	Male: 19
	Female: 6
Causes of nephrectomy	Renal cell carcinoma: 22
	Ureteral carcinoma: 2
	Metastasis in kidney: 1
Past history	Heart disease: 1
	Malignancy: 3
	Others: 15
Comorbidity	Heart disease: 6
	Cerebrovascular disease: 1
	Malignancy: 1
	Hypertension: 10
	Diabetes mellitus: 7
	Others: 15
eGFR (ml/min/1.73m^2^)	eGFR ≥ 90: 3
	60 ≤ eGFR < 90: 6
	30 ≤ eGFR < 60: 6
	15 ≤ eGFR < 30: 1
	eGFR < 15: 9
Use of antihypertensives	RAS blockers: 7
	Diuretics: 2
	Others: 7
Height (m)	164.3±8.7
Body weight (kg)	61.6±12.7
BMI (kg/m^2^)	23.2±3.8
Systolic BP (mmHg)	135.6±20.0
Diastolic BP (mmHg)	78.8±11.3
Heart rate (/min)	72.7±13.4
PRA (ng/ml/hr)	-0.011±0.33
Plasma AngII (pg/ml)	15.9±11.4
Plasma s(P)RR (ng/ml)	24.36±7.13
Interstitial fibrosis (%)	48.9±39.8

Abbreviations: eGFR, estimated glomerular filtration rate; BMI, body mass index; BP, blood pressure; PRA, plasma renin activity; AngII, angiotensin II; s(P)RR, soluble (pro)renin receptor.

### Relationships between plasma s(P)RR levels and other parameters in all the patients

We evaluated the correlation between plasma s(P)RR and other parameters in all the patients. Plasma s(P)RR levels were not correlated with age, height, body weight, body mass index (BMI), BPs, heart rate, PRA, and plasma AngII. However, significant positive relationship was found between plasma s(P)RR levels and the extent of interstitial fibrosis (Table 2).

**Table 2:** Correlations between plasma soluble (pro)renin receptor [s(P)RR] and clinical parameters in all the patients.

	Correlation coefficient	p value
Age (year)	0.039	0.85
Height (m)	0.093	0.66
Body weight (kg)	0.33	0.11
BMI (kg/m^2^)	0.35	0.093
Systolic BP (mmHg)	0.16	0.46
Diastolic BP (mmHg)	0.092	0.66
Heart rate (/min)	-0.19	0.37
PRA (ng/ml/h)	0.18	0.41
Plasma AngII (pg/ml)	0.39	0.056
Interstitial fibrosis (%)	0.52	0.008

Abbreviations: BMI, body mass index; BP, blood pressure; PRA, plasma renin activity; AngII, angiotensin II.

### Multiple linear regression analyses for plasma s(P)RR levels in all the patients

To evaluate the relationship between plasma s(P)RR levels and the extent of interstitial fibrosis, hence the contribution of s(P)RR to tubulointerstitial damage, we performed multiple linear regression analyses, after adjusting for age, sex, body weight, systolic BP, and plasma AngII levels (Table 3). We selected age, sex, and body weight as variables, because these parameters are common in performing multiple linear regression analyses. In addition, systolic BP and plasma AngII were also adjusted for, as they are associated with renal damage. All multiple regression equations revealed high prediction accuracy and significance. The analyses revealed that plasma s(P)RR was associated with the levels of interstitial fibrosis when age, sex, body weight, systolic BP, and plasma AngII were adjusted as independent variables.

**Table 3:** Multiple regression analyses for plasma soluble (pro)renin receptor [s(P)RR] in all the patients.

	Model 1		Model 2		Model 3	
	R = 0.62	p = 0.049	R = 0.71	p = 0.019	R = 0.76	p = 0.011
	β	p value	β	p value	β	p value
Age (year)	0.057	0.78	0.16	0.40	0.18	0.33
Sex	0.15	0.46	0.16	0.39	0.21	0.24
Body weight (kg)	0.38	0.079	0.48	0.024	0.51	0.013
Systolic BP (mmHg)			-0.50	0.049	-0.38	0.13
Plasma AngII (pg/ml)					0.31	0.086
Interstitial fibrosis (%)	0.51	0.012	0.83	0.002	0.71	0.006

BP, blood pressure; AngII, angiotensin II.

### Characteristics of patients not on RAS blocker therapy

Because RAS blockers influence both systemic and intrarenal RAS expression levels [12, 15, 16], we excluded 7 patients who were prescribed RAS blockers (i.e., angiotensin II receptor blockers, angiotensin-converting enzyme inhibitors, mineralocorticoid receptor blockers, or direct renin inhibitors), and their characteristics are listed in S1 Table. Of the 5 patients, those with an eGFR < 15 mL/min/1.73m^2^ underwent dialysis (HD, 4; PD, 1).

### Relationships between plasma s(P)RR levels and other parameters in the patients without RAS blockers

We evaluated the correlation between plasma s(P)RR and other parameters in the patients not on RAS blocker therapy. Plasma s(P)RR levels were not correlated with age, height, BP, heart rate, and circulating RAS. However, we found significant positive relationships between plasma s(P)RR and body weight, BMI or the extent of interstitial fibrosis (S2 Table).

### Multiple linear regression analyses for plasma s(P)RR levels for patients not on RAS blockers

We performed multiple linear regression analyses to assess the correlation between s(P)RR levels and extent of interstitial fibrosis and to evaluate the contribution of s(P)RR toward tubulointerstitial damage, for patients not on RSA blockers (S3 Table). All multiple regression equations revealed high prediction accuracy and significance. The analyses revealed an association between plasma s(P)RR and the extent of interstitial fibrosis similar to that found in all the patients.

### Intrarenal (P)RR protein expression regions and levels

**Fig 2:**
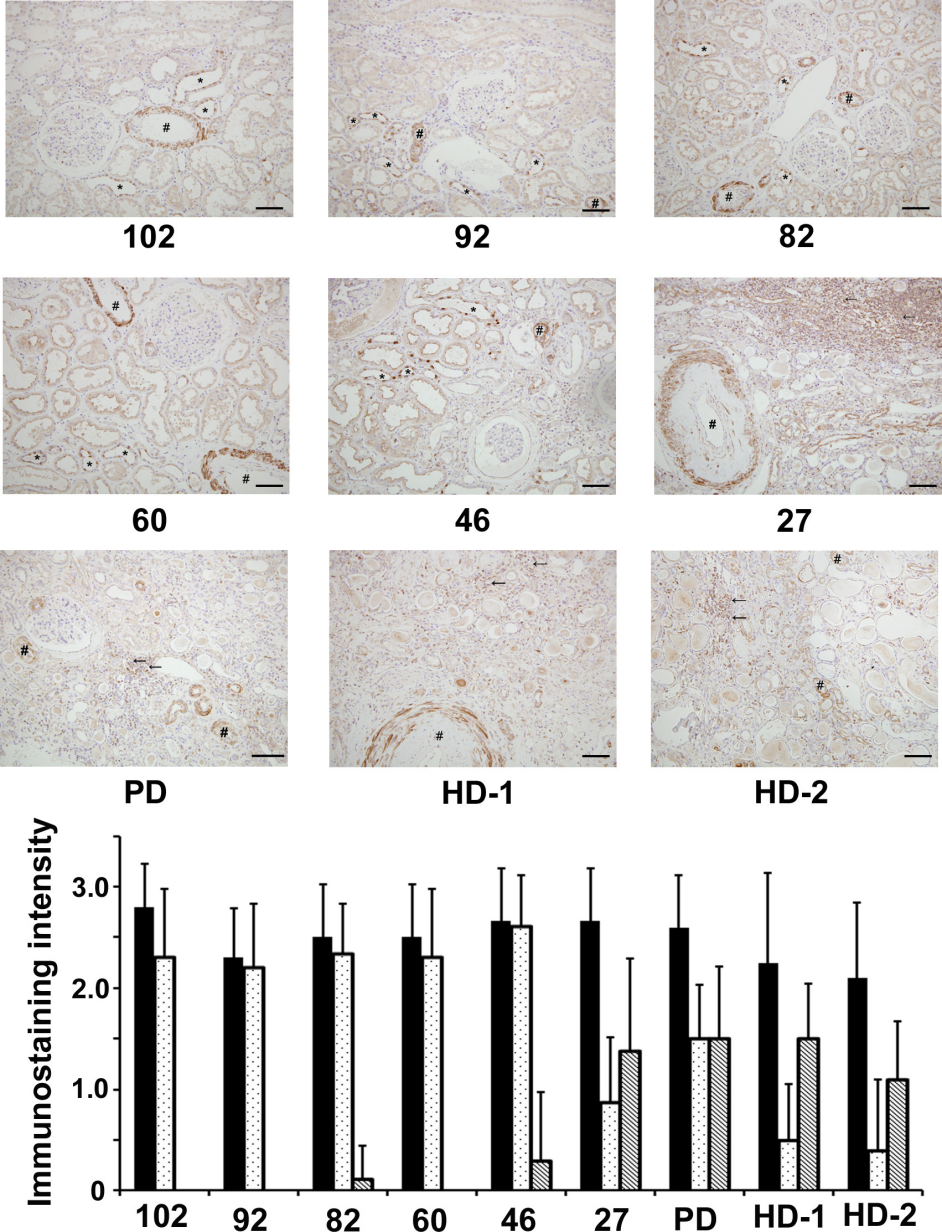
Immunostaining for (pro)renin receptor [(P)RR]. Ten small vessels and collecting ducts or connecting tubular cells and infiltrated cells in 10 microscopic fields at ×100 magnification were evaluated for representative samples according to the stained levels for (P)RR: unstained, 0; weak, 1; moderate, 2; and severe, 3; and mean values were calculated. There were no significant differences among patients in the levels of immunostaining in the small vessels. The levels of immunostaining in the collecting ducts or connecting tubular cells for the patients who had worse renal function were weaker than those who had better renal function. On the other hand, more infiltrated cells for (P)RR positive were found for patients who had the worse renal function, compared with those who had better renal function. Sharps (#), asterisks (*), and arrows (←) indicate the vessels, the collecting ducts or connecting tubule, and infiltrating cells positive for (P)RR, respectively. Numbers below each figure mean estimated glomerular filtration rate of each patient. Original magnification ×200. The scale bar in each figure represents 100 μm. The same patients in whom Masson’s trichrome staining were performed were selected. Black, dotted, and diagonal bars indicate the immunostaining intensity levels of small vessels, collecting ducts or connecting tubular cells, and infiltrated cells, respectively. Abbreviations: PD, peritoneal dialysis; HD, hemodialysis

Immunostaining for (P)RR was observed to clarify the expression regions and levels. The levels of immunostaining were weaker in the collecting ducts or connecting tubular cells of the patients who had worse renal function, including patients on dialysis, than those with better renal function. Immunostaining results for small vessels did not significantly differ among the patients. However, mononuclear cell infiltration was prominent in patients with poor renal function, such as those on dialysis, compared with those with better renal function, and some of the infiltrated cells were positive for (P)RR (Fig 2).

### Staining of infiltrated cells by using (P)RR and cell surface markers in serial sections and double staining of (P)RR and cell surface markers

**Fig 3:**
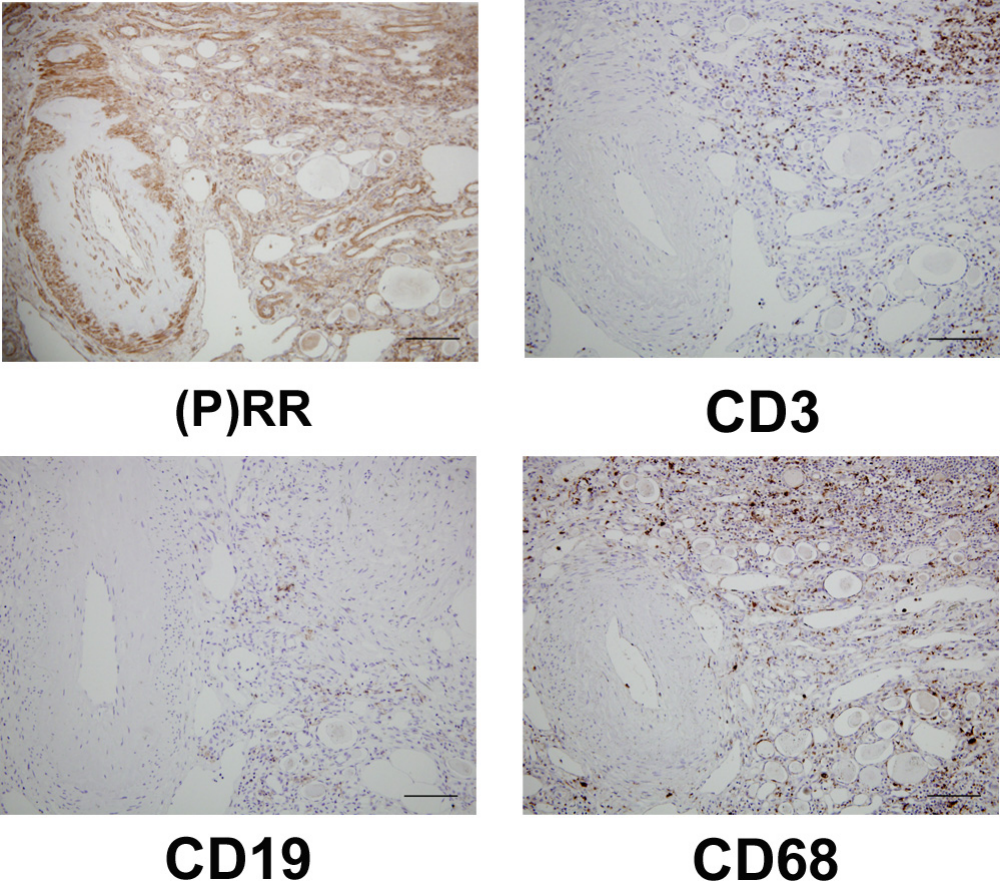
Staining of infiltrated cells by using (pro)renin receptor ((P)RR) and cell surface markers in serial sections. (P)RR was positive for vessels, collecting ducts or connecting tubules, and infiltrated cells. Most of infiltrated cells positive for (P)RR were CD3-positive cells (T cell line), and CD19-positive cells (B cell line) were sparse. CD68 positive cells (monocyte/macrophage line) were diffusely scattered. Original magnification ×200. The scale bar in each figure represents 100 μm. The patient whose eGFR was 27 mL/min/1.73m^2^ was selected for the immunohistochemical analyses because remarkable infiltrated cells were present in the tissue section.

**Fig 4:**
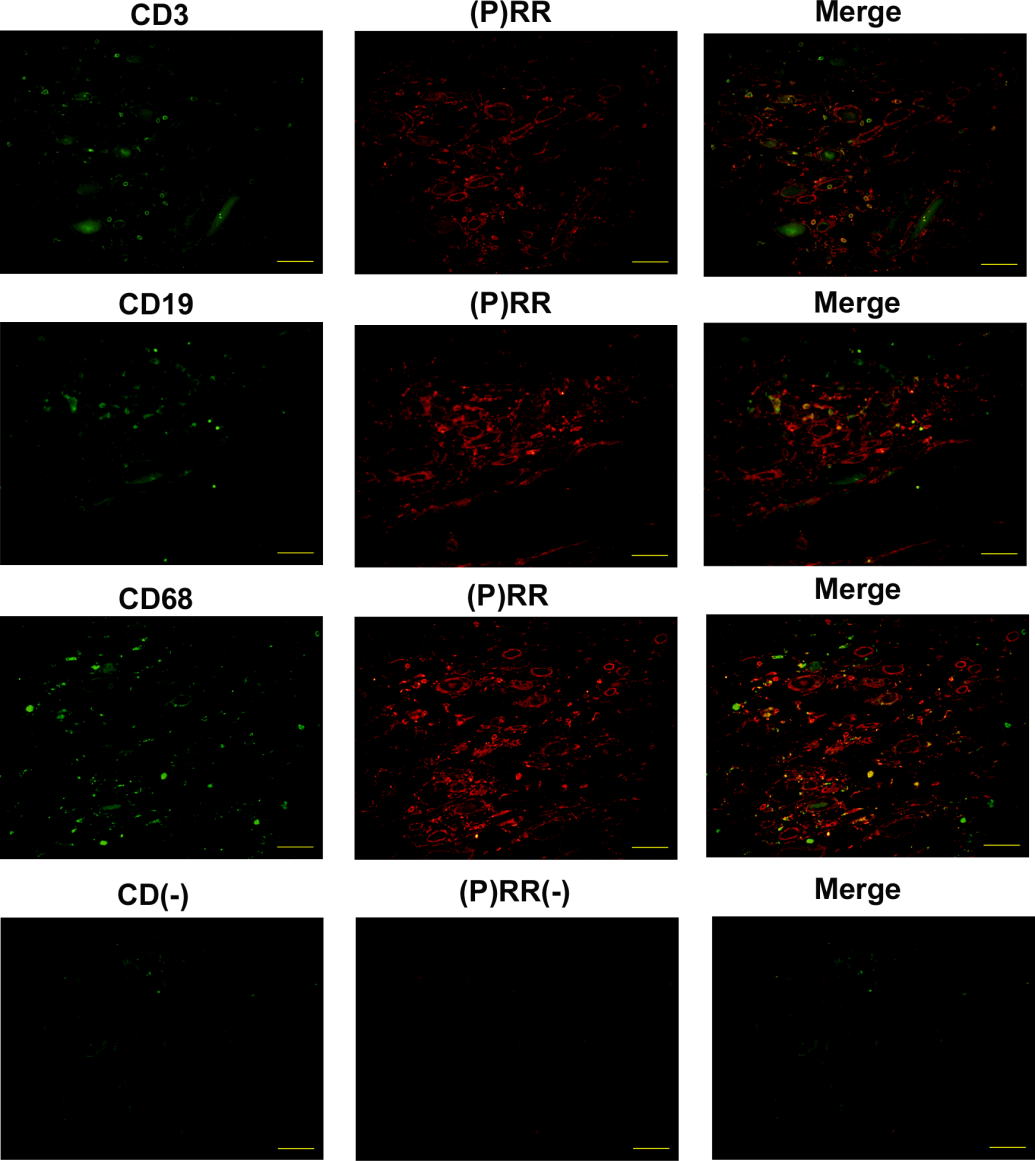
Double staining by immunofluorescence using anti-(P)RR antibody and antibodies for cell surface markers. Double staining of paraffin sections was performed using anti-(P)RR antibody with either anti-CD3, anti-CD19, or anti-CD68 antibody, respectively. (P)RR and CD3 were merged well. A few merged cells of (P)RR and CD68 were found in the immunofluorescence study. On the other hand, it was difficult to determine the merged cells of (P)RR and CD19 clearly. Original magnification ×400. The scale bar in each figure represents 100 μm. The patient whose eGFR was 27 mL/min/1.73m^2^ was selected for the immunofluorescence analyses.

Staining of infiltrated cells using (P)RR and cell surface markers in serial sections and double staining of (P)RR and cell surface markers were performed to determine what kinds of cells were infiltrated. Most of the infiltrated cells positive for (P)RR were CD3-positive cells (T cell line), and (P)RR and CD3 were merged well. CD19-positive cells (B cell line) were sparse in infiltrated cells, and it was difficult to determine the merged cells clearly. CD68-positive cells (monocyte/macrophage cell line) were diffusely scattered, and a few merged cells were found in the immunofluorescence study (Figs 3 and 4).

## Discussion

In this study, significant positive relationships were found between plasma s(P)RR levels and levels of tubulointerstitial fibrosis in both all the patients and the patients without RAS blockers. These relationships were maintained even after adjustment for age, sex, body weight, plasma AngII levels, a surrogate marker of circulating RAS, and systolic BP in all the patients and the patients without RAS blockers. These data suggest that plasma s(P)RR can be a surrogate marker for renal damage.

It is not clear why plasma s(P)RR reflects renal damage in this study. A previous report demonstrated increased expression levels of (P)RR in the remnant kidneys of 5/6 nephrectomized rats [17]. Ichihara et al. provided direct evidence that (P)RR blockers could significantly prevent and suppress end-stage kidney disease [18]. In addition, renal damage upregulates renal RAS activity, and subsequently, increases renal AngII. Increased renal AngII stimulates inflammation and fibrosis in the renal interstitial region due to enhanced oxidative stress through an increase in NADPH oxidase activity [19] and elevation of transforming growth factor-β1 expression levels [20]. Moreover, increased renal AngII induces interstitial macrophage infiltration due to augmentation of monocyte chemotactic protein-1 [21]. Most infiltrated cells are positive for (P)RR as indicated in this study, and s(P)RR is cleaved from (P)RR in the cells and is secreted and ultimately found in the blood. In this way, it is possible that s(P)RR is the surrogate marker of intrarenal fibrosis.

Three components (small vessels, collecting ducts or connecting tubules, and infiltrated cells) were positive for (P)RR in this study. Immunostaining results for small vessels did not significantly differ among the patients, and the levels of immunostaining were weaker in the collecting ducts and connecting tubular cells of the patients who had worse renal function than those with better renal function in this study. Recently, it has been shown that (P)RR is expressed in human lymphocytes and monocytes and that (P)RR contributes to inflammation [22]. Because we did not separate peripheral blood mononuclear cells and analyze the expression levels of (P)RR using polymerase chain reaction or immunoblotting, we could not quantify (P)RR expression levels in the infiltrated cells precisely. However, more infiltrated cells were found, and some of the infiltrated cells were positive for (P)RR more frequently according to the renal dysfunction. In addition, we determined that most of the infiltrated cells positive for (P)RR were CD3-positive (T cell line), and some of them were CD68-positive cells (monocyte/macrophage cell line), and CD19-positive cells (B cell line) were not evident. These results may suggest that plasma s(P)RR was supplied from the infiltrated (P)RR-positive T cells or monocytes/macrophages that coincide with the previous report [22]. However, s(P)RR levels are affected by many factors, including RAS blocker administration [23], lipid metabolites such as high-density lipoprotein cholesterol and triglycerides [24], age [24], and obstructive sleep apnea syndrome [25]. Moreover, the correlation between s(P)RR level and furin activity has not yet been investigated. Further investigations are expected to determine the mechanism of s(P)RR generation and the contribution of s(P)RR for renal damage.

The significant positive relationships between plasma s(P)RR levels and renal damage were obtained in all the patients in this study. Because previous reports showed that angiotensin receptor blockers significantly inhibit renal expression of (P)RR mRNA and protein in diabetic rats and patients with CKD [8, 26], we reanalyzed the relationship between plasma s(P)RR levels and renal damage, using only the patients without RAS treatment, and the significant positive relationships between plasma s(P)RR levels and renal damage were also obtained in the patients without RAS treatment similar to all the patients. These results strongly indicate the importance of plasma s(P)RR for renal damage.

Significant positive relationship between sCr levels and s(P)RR levels and significant negative relationship between eGFR levels and s(P)RR levels have been previously reported [8, 24]. It is likely that the relationship between s(P)RR and tubulointerstitial fibrosis levels was influenced by renal function in this study. However, the purpose of this study was to clarify that plasma s(P)RR levels reflected renal damage for all the patients, including patients on dialysis. Adjustment for renal function was not performed in this study because eGFR was not applicable to patients on dialysis.

We have investigated the relationship between urinary albumin and protein excretion that is a surrogate marker of renal damage and s(P)RR. Contrary to expectations, no significant relationships were found between them (r = 0.088, p = 0.76). The following are the possible causes: (1) eight hemodialysis patients were excluded because of anuria, and the sample size became much smaller. (2) The urinary protein excretion levels that could be measured from the remaining patients were 237.8±307.6 mg/day, and it is supposed that the causes of renal damage in these patients were not glomerulopathies with proteinuria.

Because (P)RR is widely expressed in various tissues, including the kidney, heart, liver, placenta, and pancreas, it is possible that increased s(P)RR came from other tissues but not the kidney. However, no tissue is injured in other tissues. Therefore, it is less likely that infiltrated cells positive for (P)RR came from other tissues. However, to clarify the origin of s(P)RR, the relationship between tissue-specific (P)RR expression levels and expression levels in the kidney is needed to be investigated using tissue-specific (P)RR knockout mouse. In the future, we would like to perform these experiments.

In summary, significant positive relationships were found between plasma s(P)RR levels and levels of tubulointerstitial fibrosis in both all the patients and the patients without RAS blocker therapy. These relationships were maintained even after adjustment for age, sex, body weight, plasma AngII, and systolic BP. In addition, (P)RR expression was elevated in infiltrated mononuclear cells but not in connecting tubules or collecting ducts and vessels. These data suggest that plasma s(P)RR that may reflect (P)RR expression levels in infiltrated mononuclear cells can be a surrogate marker for renal damage.

## Supporting Information

S1 Table Characteristics of patients without renin-angiotensin system (RAS) blockers. (DOC)

S2 Table Correlations between plasma soluble (pro)renin receptor [s(P)RR] and clinical parameters in patients without renin-angiotensin system (RAS) blockers. (DOC)

S3 Table Multiple regression analyses for plasma soluble (pro)renin receptor [s(P)RR] in patients without renin-angiotensin system (RAS) blockers. (DOC)
