# Supplementary material for: Plasma Soluble (Pro)renin Receptor Reflects Renal Damage
Source: PLoS One. 2016 May 26;11(5):e0156165. doi: 10.1371/journal.pone.0156165 (PMC4881895; doi:10.1371/journal.pone.0156165)
Supplement: S1 Table — (DOC) [file pone.0156165.s001.doc]

**Supplementary Table 1**: Characteristics of patients without renin-angiotensin system (RAS) blockers

| Age, year | 63.1 ± 16.3 |
| --- | --- |
| Sex | Male: 13 |
|  | Female: 5 |
| Causes of nephrectomy | Renal cell carcinoma: 16 |
|  | Ureteral carcinoma: 1 |
|  | Metastasis in kidney: 1 |
| Past history | Diabetes mellitus: 1 |
|  | Malignancy: 2 |
|  | Others: 12 |
| Comorbidity | Heart disease: 3 |
|  | Malignancy: 1 |
|  | Hypertension: 4 |
|  | Diabetes mellitus: 6 |
|  | Others: 10 |
| eGFR (ml/min/1.73m^2^) | eGFR ≥ 90: 3 |
|  | 60 ≤ eGFR < 90: 5 |
|  | 30 ≤ eGFR < 60: 4 |
|  | 15 ≤ eGFR < 30: 1 |
|  | eGFR < 15 : 5 |
| Use of antihypertensives | Diuretics: 0 |
|  | Others: 5 |
| Height (m) | 164.1 ± 9.1 |
| Body weight (kg) | 61.6 ± 13.6 |
| BMI (kg/m^2^) | 23.3 ± 4.2 |
| Systolic BP (mmHg) | 133.8 ± 19.3 |
| Diastolic BP (mmHg) | 78.1 ± 10.0 |
| Heart rate (/min) | 70.7 ± 12.6 |
| PRA (ng/ml/hr) | -0.086 ± 0.28 |
| Plasma AngII (pg/ml) | 13.4 ± 8.4 |
| Plasma s(P)RR (ng/ml) | 22.89 ± 7.20 |
| Interstitial fibrosis (%) | 42.2 ± 39.3 |

Abbreviations: eGFR, estimated glomerular filtration rate; BMI, body mass index; BP, blood pressure; PRA, plasma renin activity; AngII, angiotensin II; s(P)RR, soluble (pro)renin receptor
