# Supplementary material for: Plasma Soluble (Pro)renin Receptor Reflects Renal Damage
Source: PLoS One. 2016 May 26;11(5):e0156165. doi: 10.1371/journal.pone.0156165 (PMC4881895; doi:10.1371/journal.pone.0156165)
Supplement: S2 Table — (DOC) [file pone.0156165.s002.doc]

**Supplementary Table 2:** Correlations between plasma soluble (pro)renin receptor [s(P)RR] and clinical parameters in patients without renin-angiotensin system (RAS) blockers

|  | Correlation coefficient | p value |
| --- | --- | --- |
| Age (year) | 0.007 | 0.98 |
| Height (m) | 0.069 | 0.79 |
| Body weight (kg) | 0.49 | 0.046 |
| BMI (kg/m^2^) | 0.55 | 0.022 |
| Systolic BP (mmHg) | 0.29 | 0.24 |
| Diastolic BP (mmHg) | 0.22 | 0.37 |
| Heart rate (/min) | -0.31 | 0.21 |
| PRA (ng/ml/h) | 0.23 | 0.36 |
| Plasma AngII (pg/ml) | 0.22 | 0.38 |
| Interstitial fibrosis (%) | 0.62 | 0.006 |

Abbreviations: BMI, body mass index; BP, blood pressure; PRA, plasma renin activity; AngII, angiotensin II
