# Supplementary material for: Plasma Soluble (Pro)renin Receptor Reflects Renal Damage
Source: PLoS One. 2016 May 26;11(5):e0156165. doi: 10.1371/journal.pone.0156165 (PMC4881895; doi:10.1371/journal.pone.0156165)
Supplement: S3 Table — (DOC) [file pone.0156165.s003.doc]

**Supplementary Table 3:** Multiple regression analyses for plasma soluble (pro)renin receptor [s(P)RR] in patients without renin-angiotensin system (RAS) blockers

|  | Model 1 | | Model 2 | | Model 3 | |
| --- | --- | --- | --- | --- | --- | --- |
|  | R=0.78 | p=0.016 | R=0.81 | p=0.021 | R=0.82 | p=0.046 |
|  | β | p value | β | p value | β | p value |
| Age (year) | 0.073 | 0.75 | 0.12 | 0.59 | 0.13 | 0.60 |
| Sex | 0.29 | 0.17 | 0.22 | 0.31 | 0.23 | 0.31 |
| Body weight (kg) | 0.50 | 0.044 | 0.56 | 0.027 | 0.57 | 0.034 |
| Systolic BP (mmHg) |  |  | -0.35 | 0.22 | 0.068 | 0.73 |
| Plasma AngII (pg/ml) |  |  |  |  | -0.33 | 0.27 |
| Interstitial fibrosis (%) | 0.58 | 0.012 | 0.78 | 0.009 | 0.75 | 0.018 |

Abbreviations: BP, blood pressure; AngII, angiotensin II
